# Supplementary material for: Pathway representation via intrinsic structural medoids (PRISM): a structural mapping approach to clustering molecular pathways
Source: Digit Discov. 2026 Sep 11;5(10):4110–20. doi: 10.1039/d6dd00288a (PMC13580032; doi:10.1039/d6dd00288a)
Supplement: Supplementary file 1 [file DD-005-D6DD00288A-s001.pdf]

Supplementary Information (SI) for Digital Discovery.
This journal is © The Royal Society of Chemistry 2026

Supporting Information
Pathway Representation via Intrinsic Structural Medoids (PRISM): A Structural Mapping Approach to Clustering Molecular Pathways

Jherome Brylle Woody Santos,[1] Jeremy M. G. Leung,[2] Lillian T. Chong[2*], Ramón Alain Miranda-Quintana[1*]

1. Department of Chemistry and Quantum Theory Project, University of Florida, Gainesville, Florida, 32611, USA

2. Department of Chemistry, University of Pittsburgh, Pittsburgh, Pennsylvania 15260, USA

Email: ltchong@pitt.edu, quintana@chem.ufl.edu

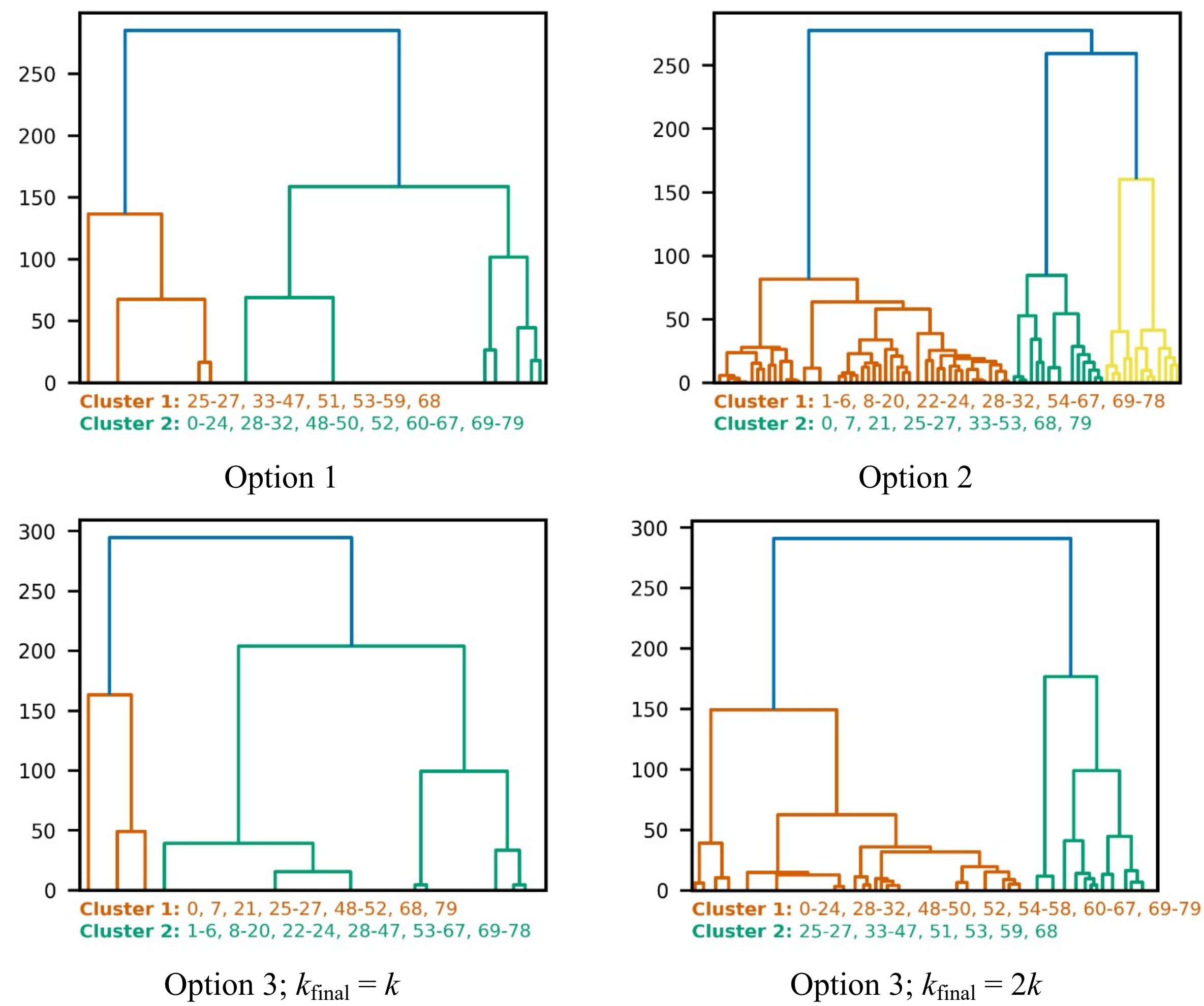

Figure S1. Alanine dipeptide pathway dendrograms for PRISM variants (Options 1, 2, 3; k=6).

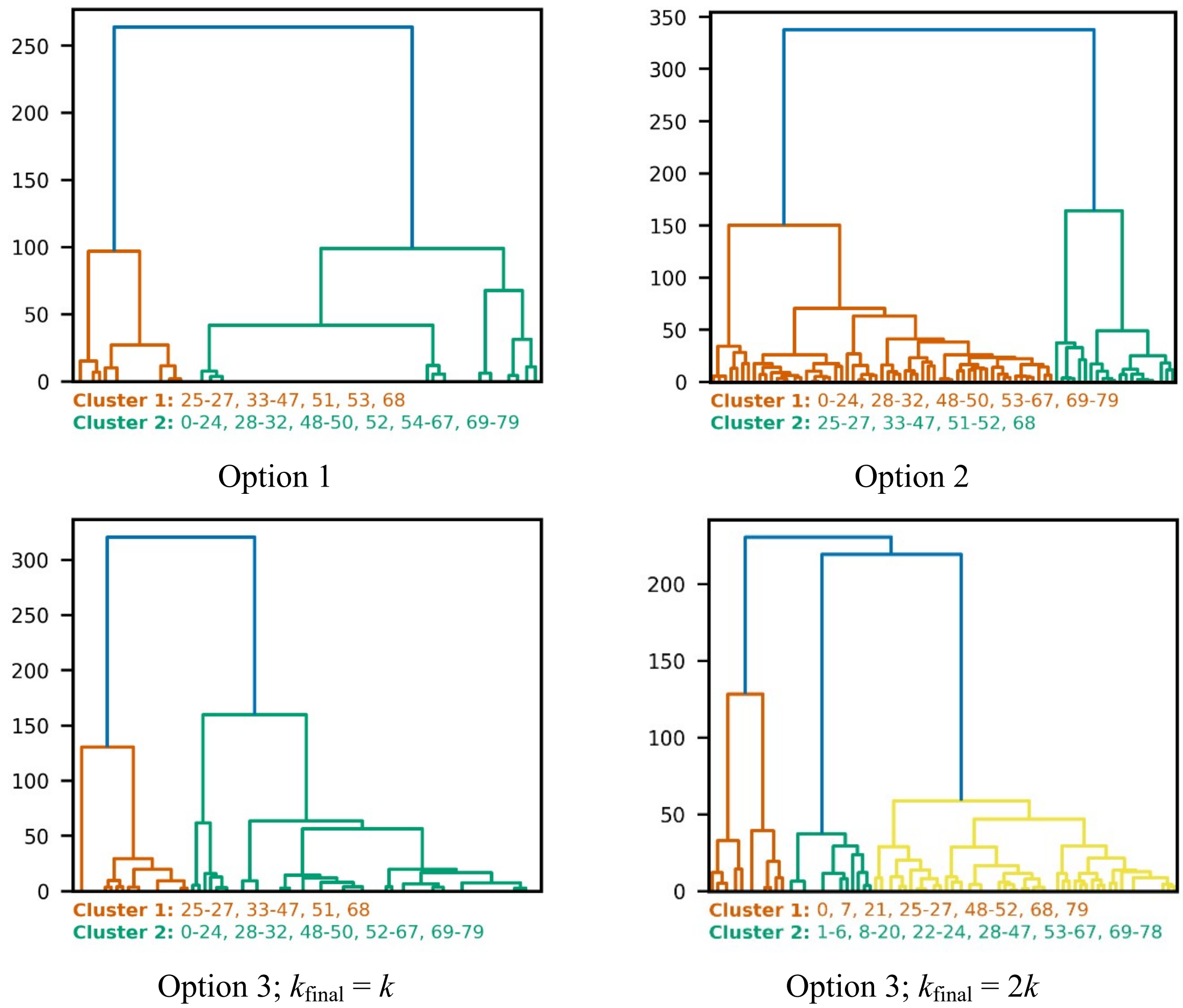

Figure S2. Alanine dipeptide pathway dendrograms for PRISM variants (Options 1, 2, 3; k=10).

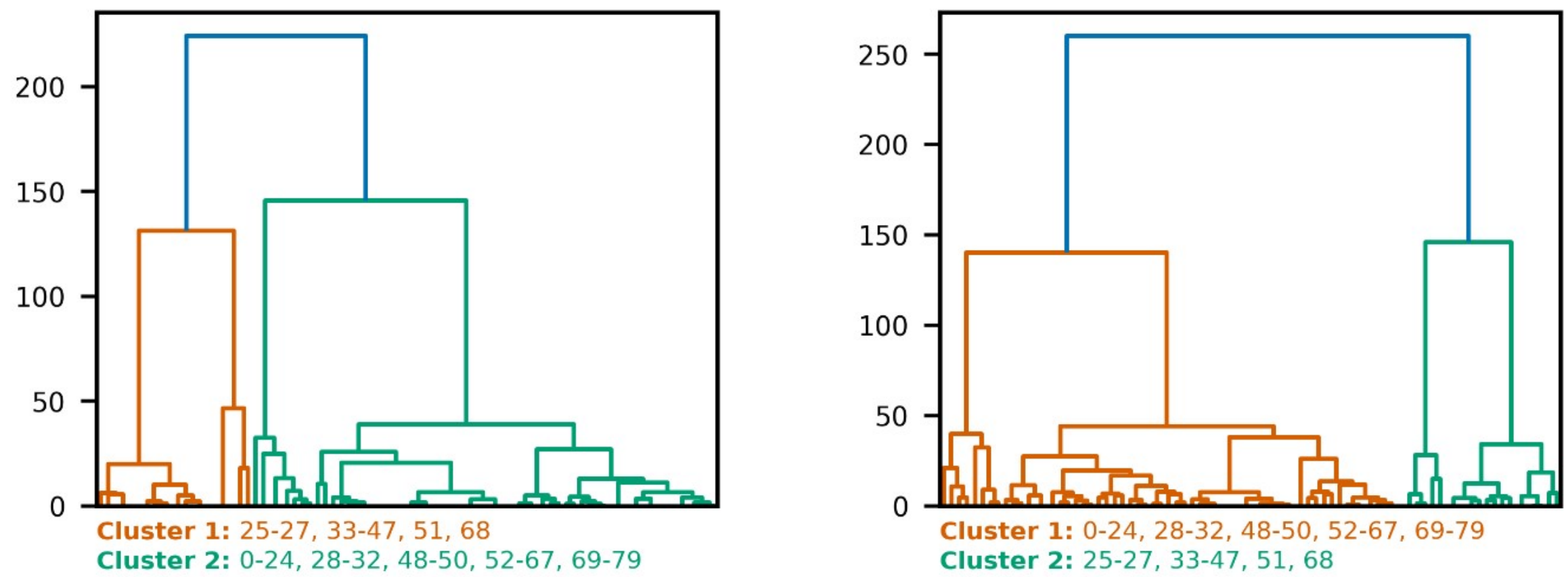

Option 3; $k_{\text{final}} = k$ Option 3; $k_{\text{final}} = 2k$

Figure S3. Alanine dipeptide pathway dendrograms for PRISM Option 3; k=20.

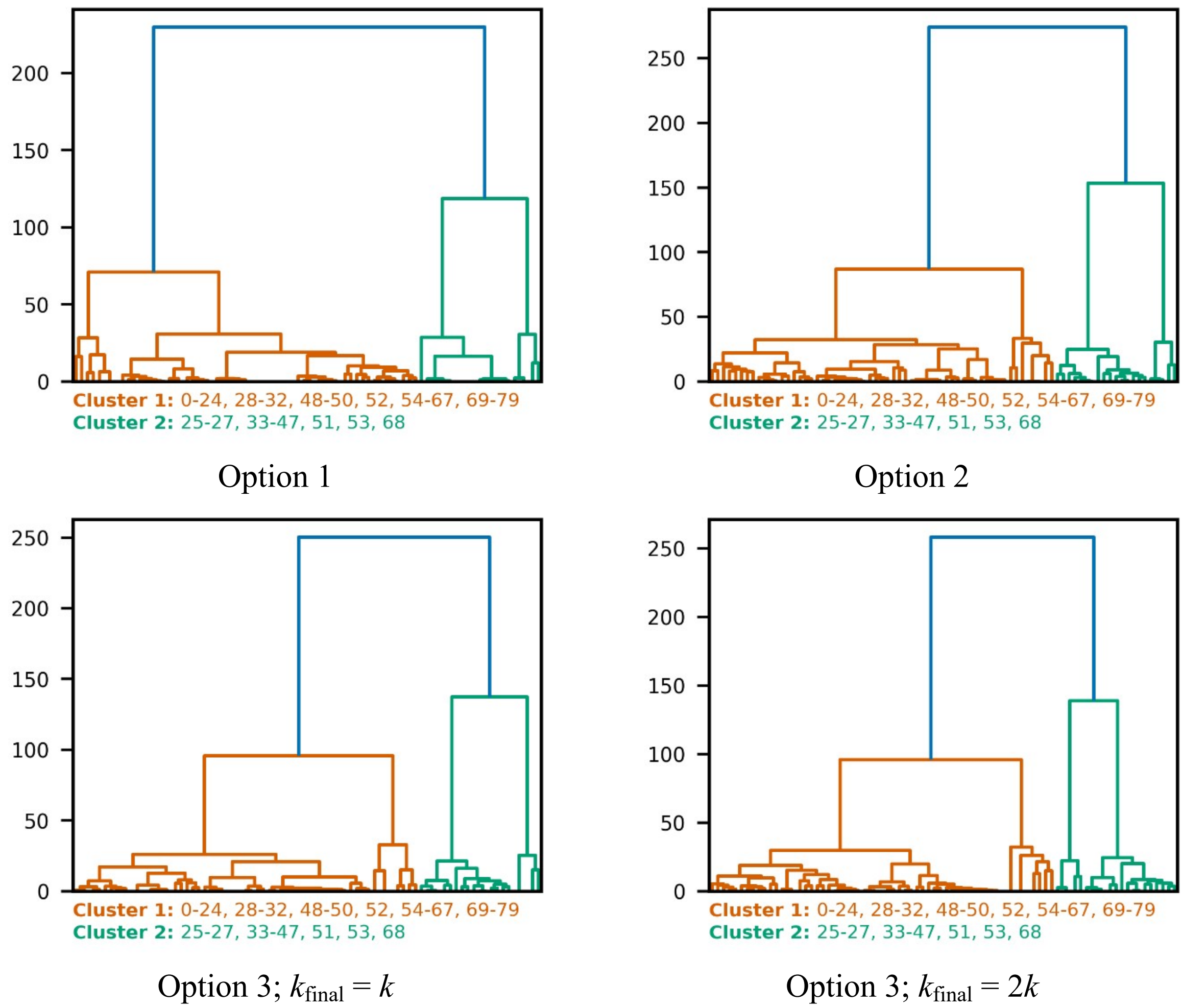

Figure S4. Alanine dipeptide pathway dendrograms for PRISM variants (Options 1, 2, 3; k=50).

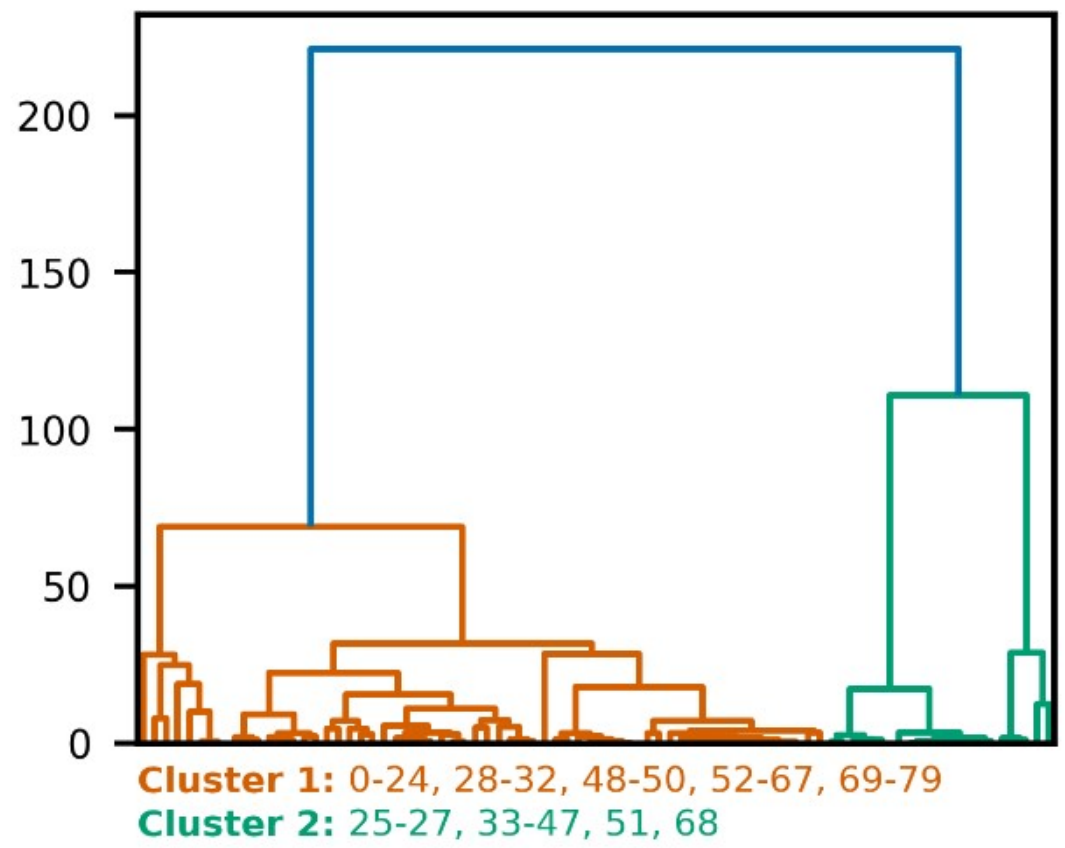

Option 1

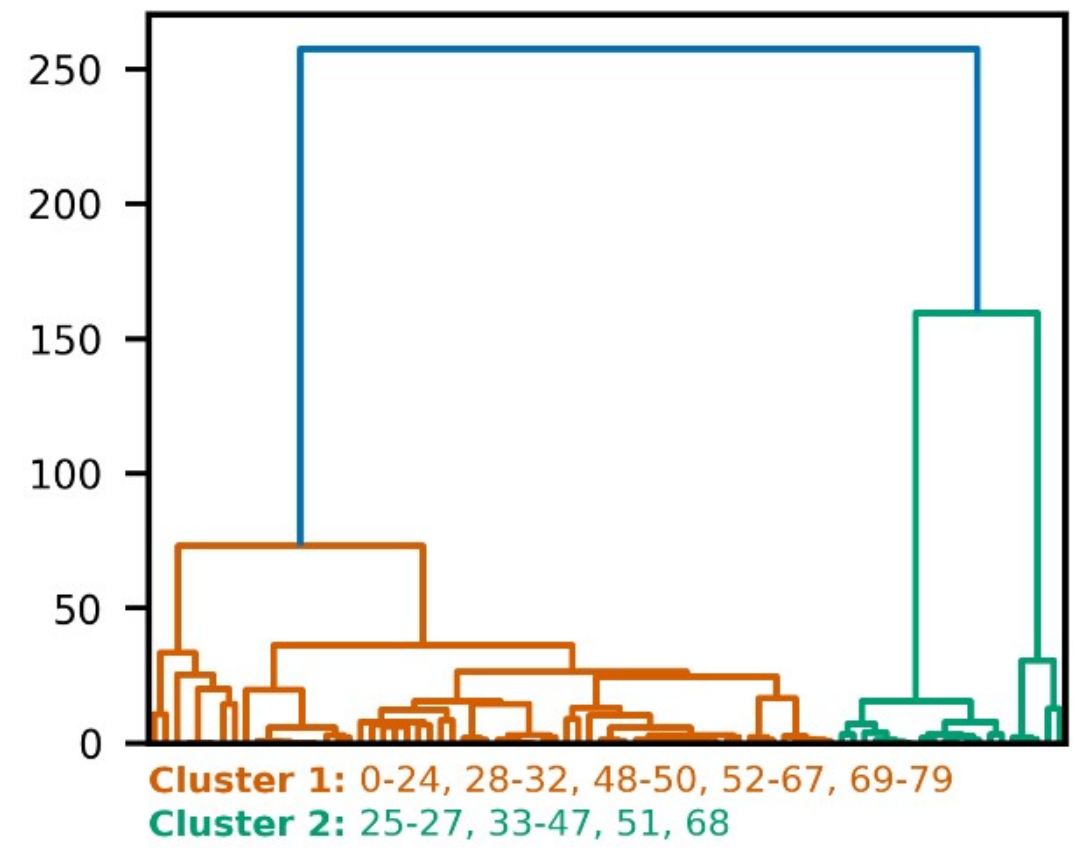

Option 2

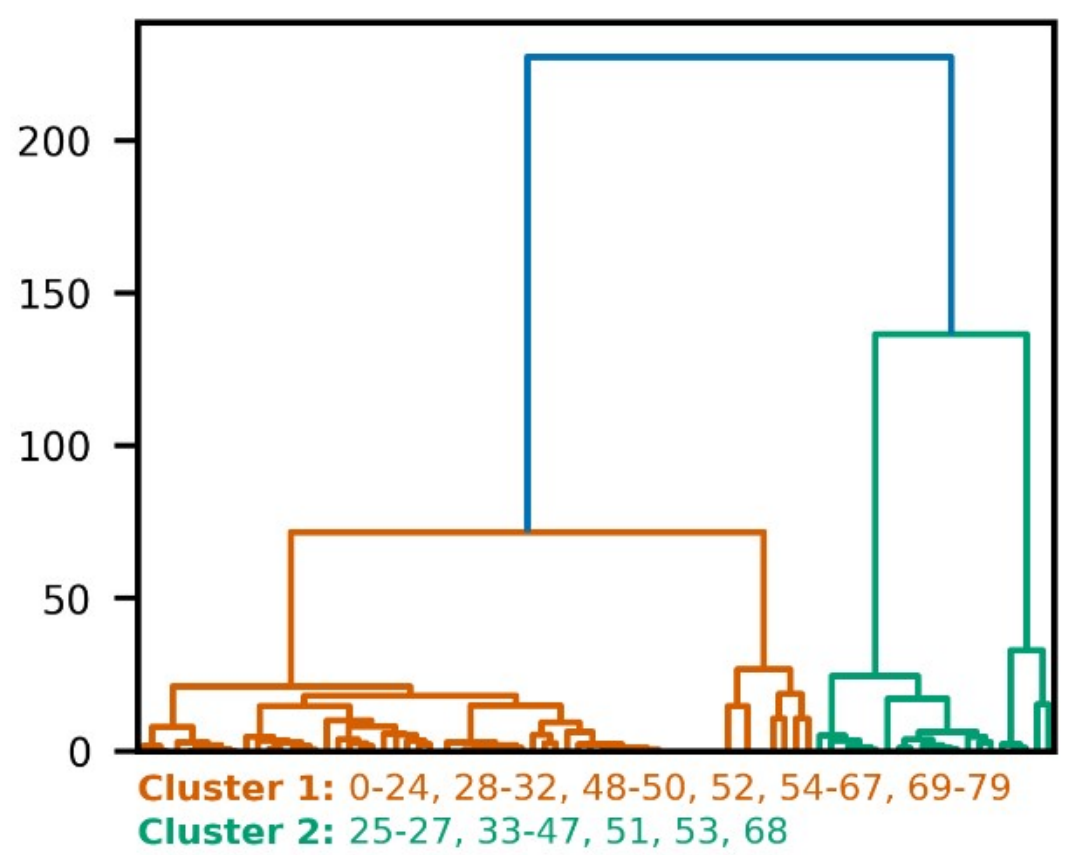

Option 3; $k_{\text{final}} = k$

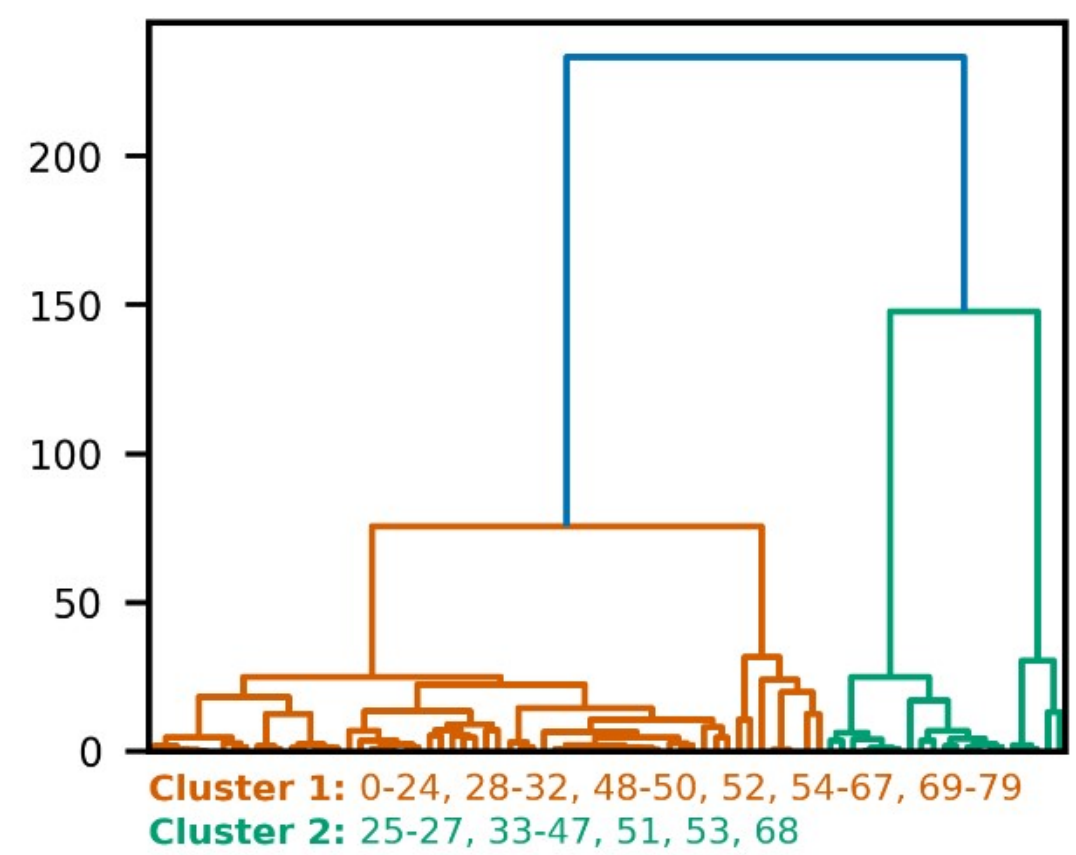

Option 3; $k_{\text{final}} = 2k$

Figure S5. Alanine dipeptide pathway dendrograms for PRISM variants (Options 1, 2, 3; *k*=80).

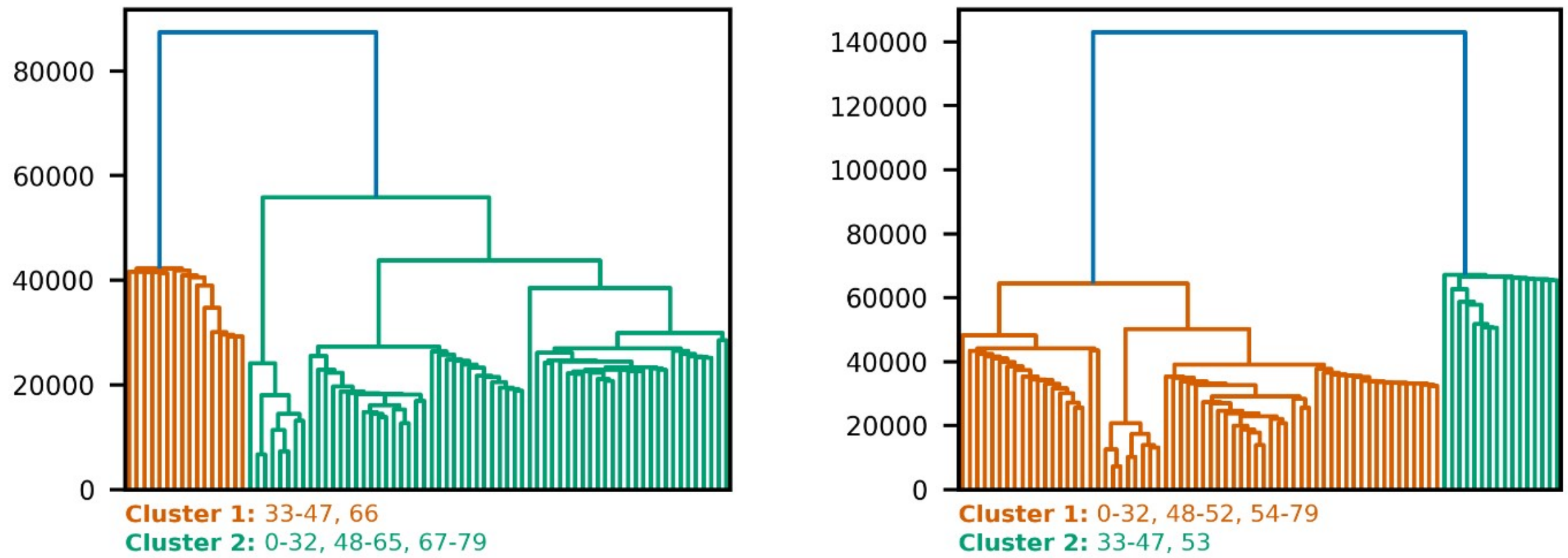

Intra; No Sampling

Intra; 50% Diversity Sampling

Figure S6. Alanine dipeptide pathway dendrograms for SHINE intra (with and without sampling).

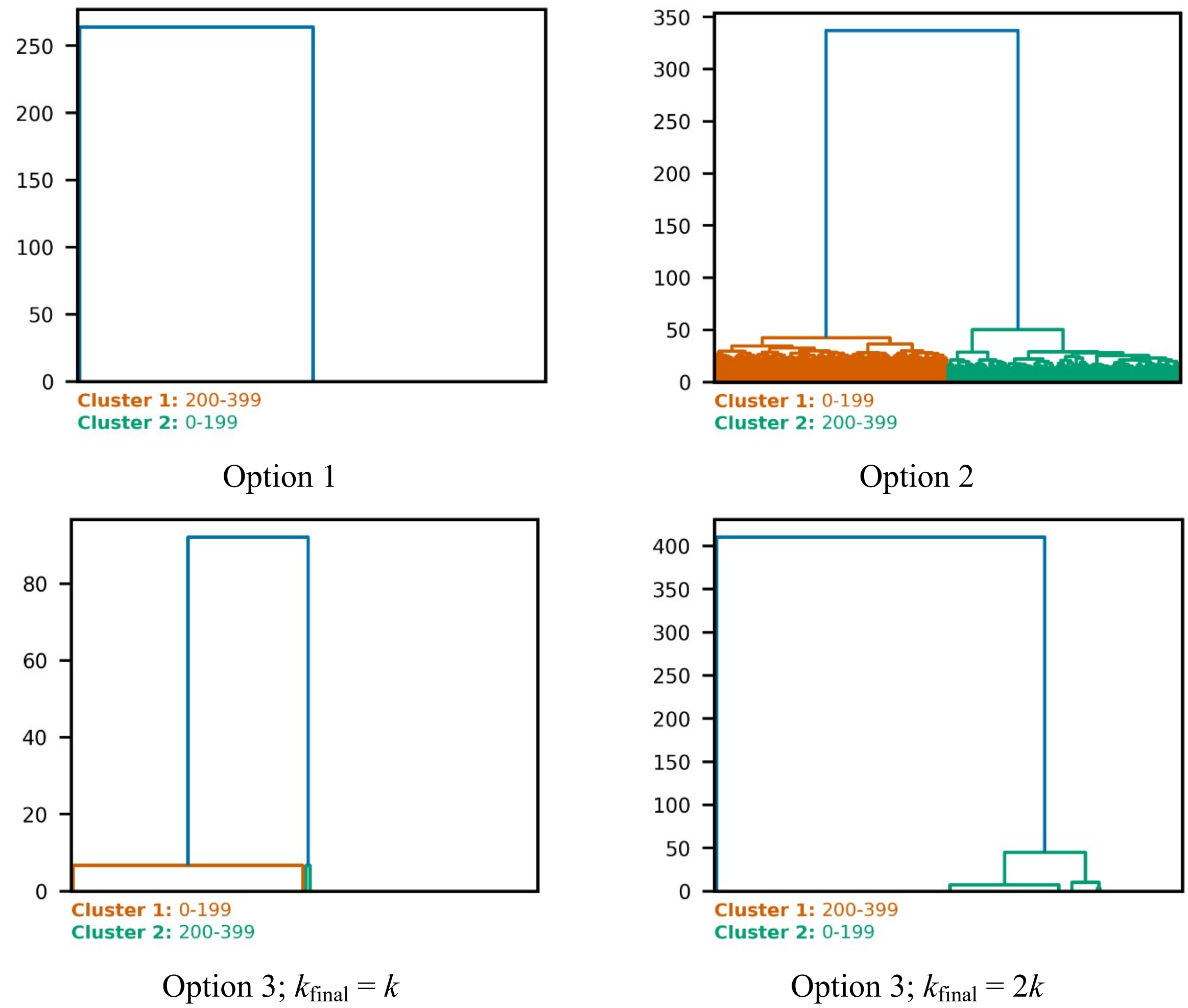

Figure S7. Adenylate kinase pathway dendrograms for PRISM variants (Options 1, 2, 3; *k*=6).

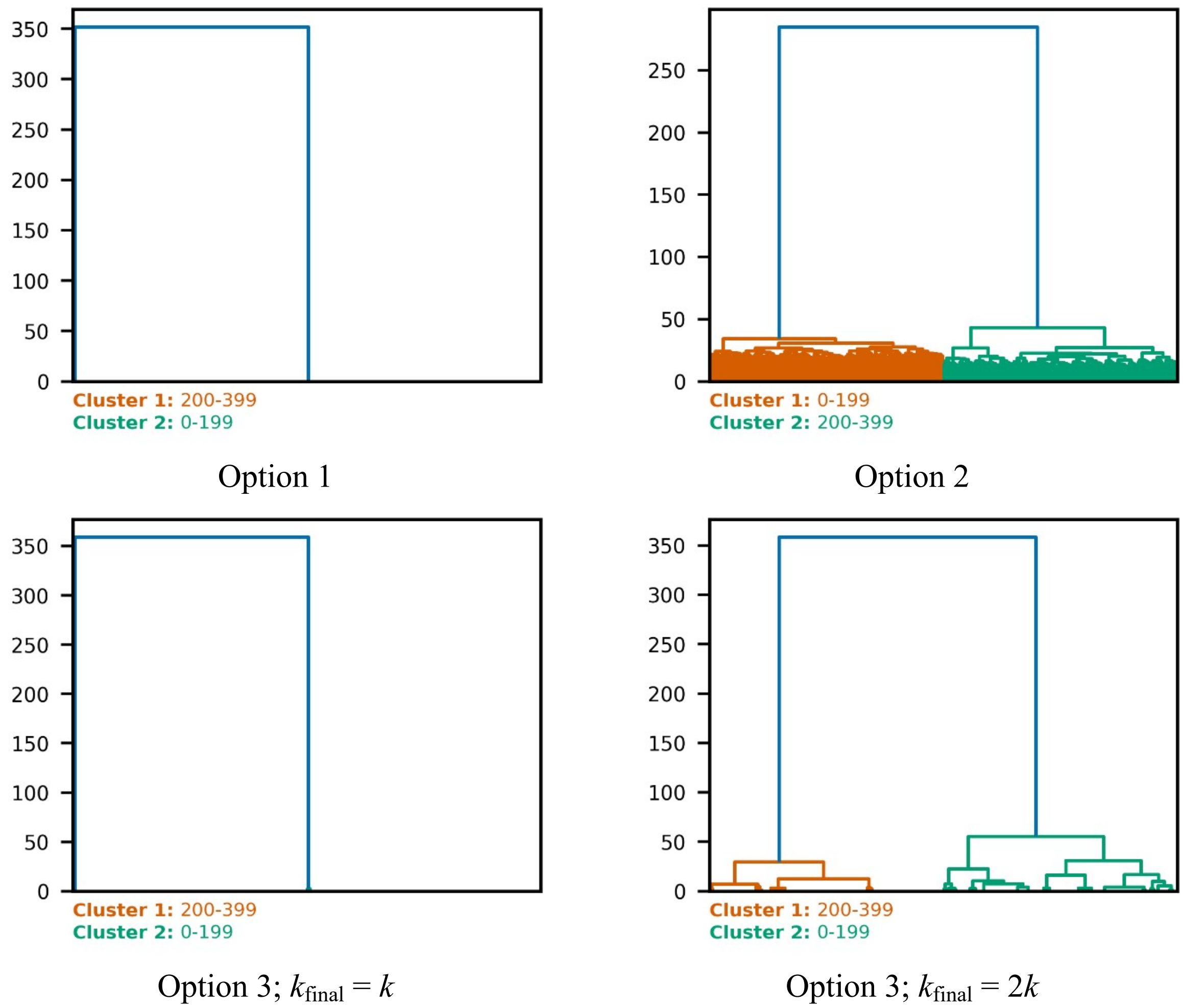

Figure S8. Adenylate kinase pathway dendrograms for PRISM variants (Options 1, 2, 3; k=10).

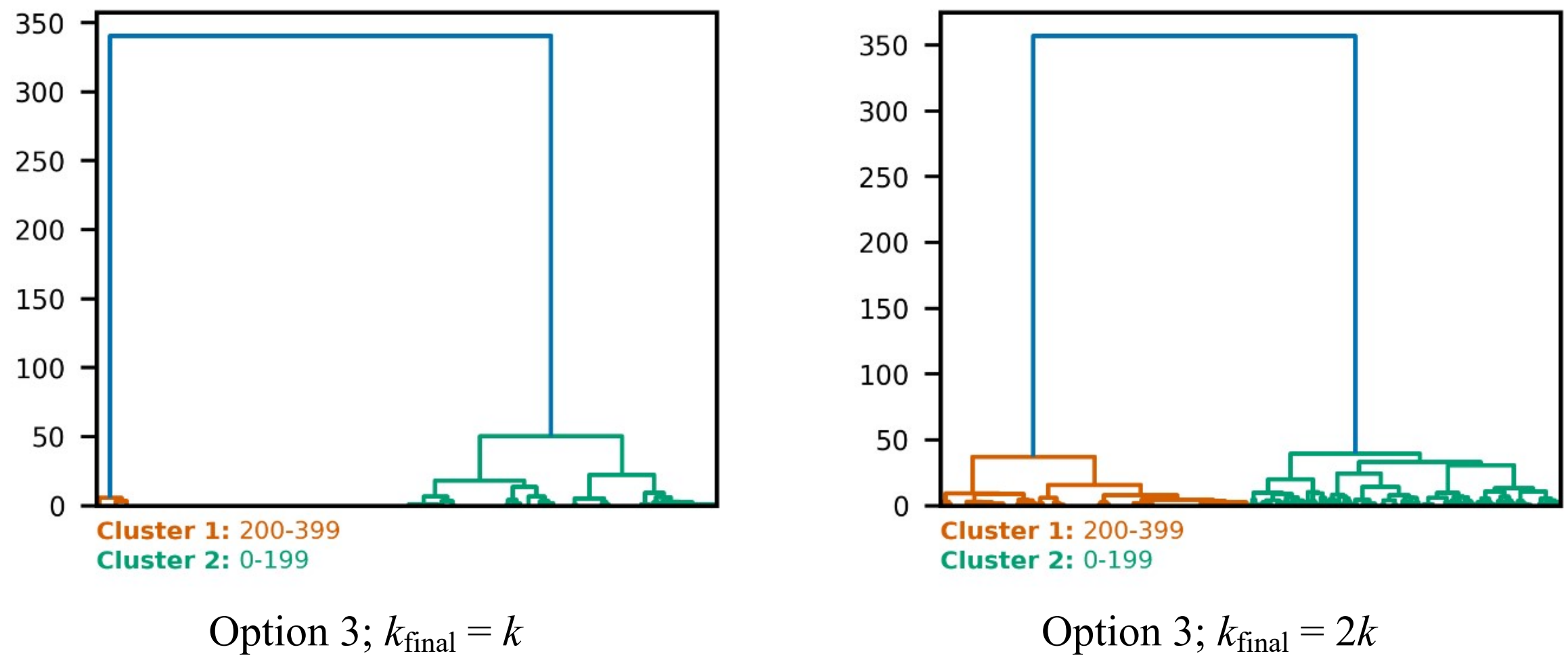

Figure S9. Adenylate kinase pathway dendrograms for PRISM Option 3; k=20.

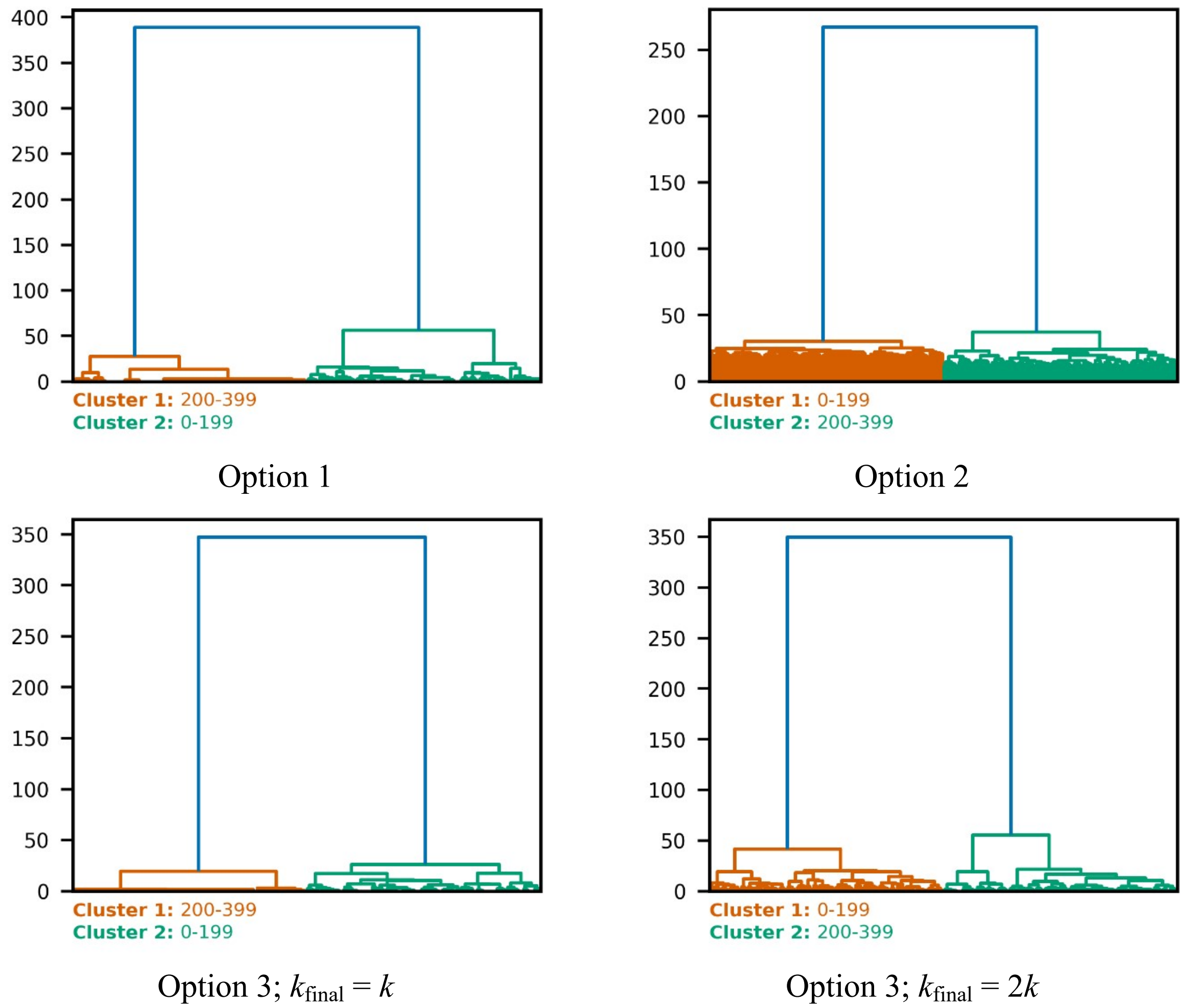

Figure S10. Adenylate kinase pathway dendrograms for PRISM variants (Options 1, 2, 3; k=50).

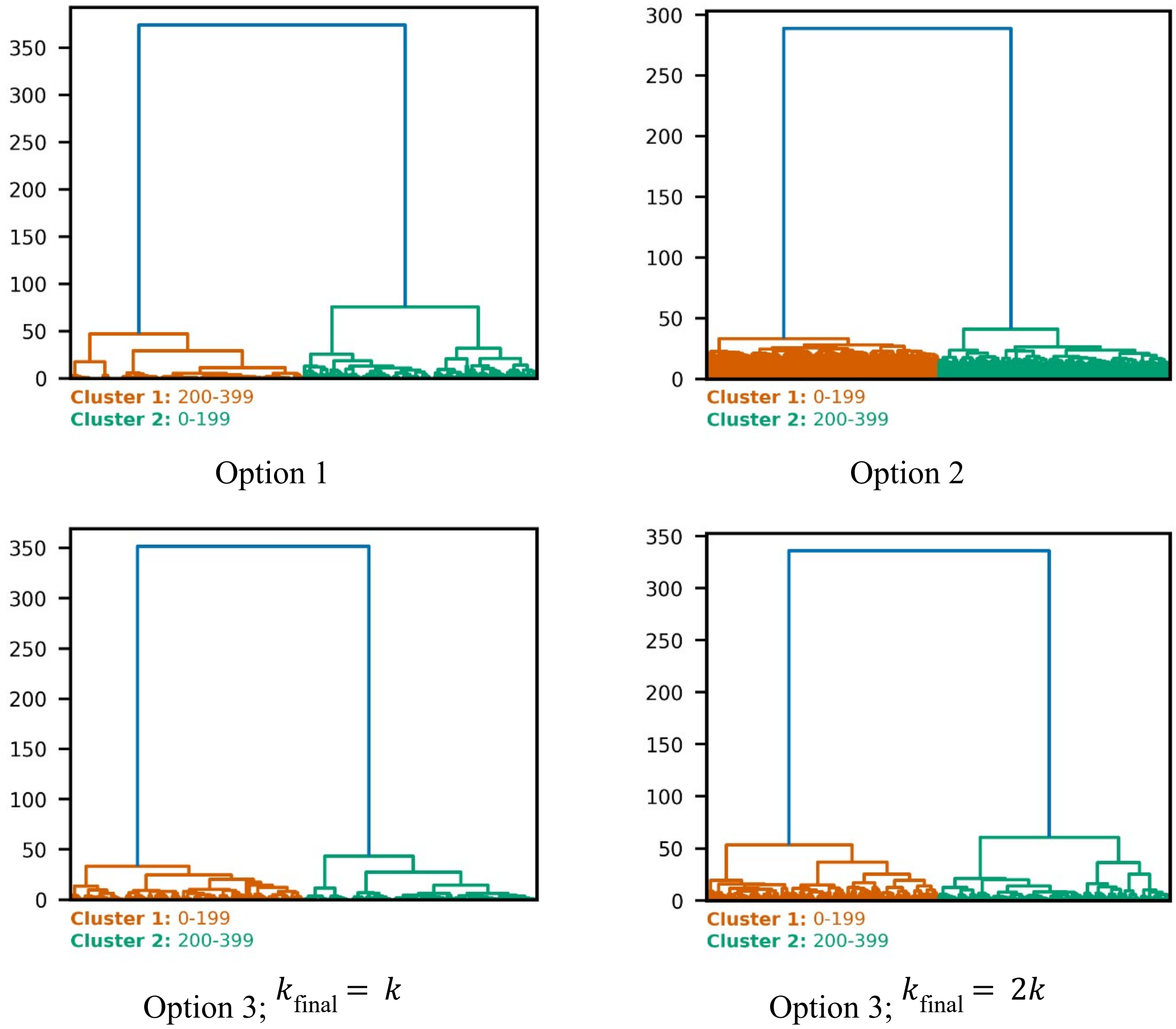

Figure S11. Adenylate kinase pathway dendrograms for PRISM variants (Options 1, 2, 3; k=80).

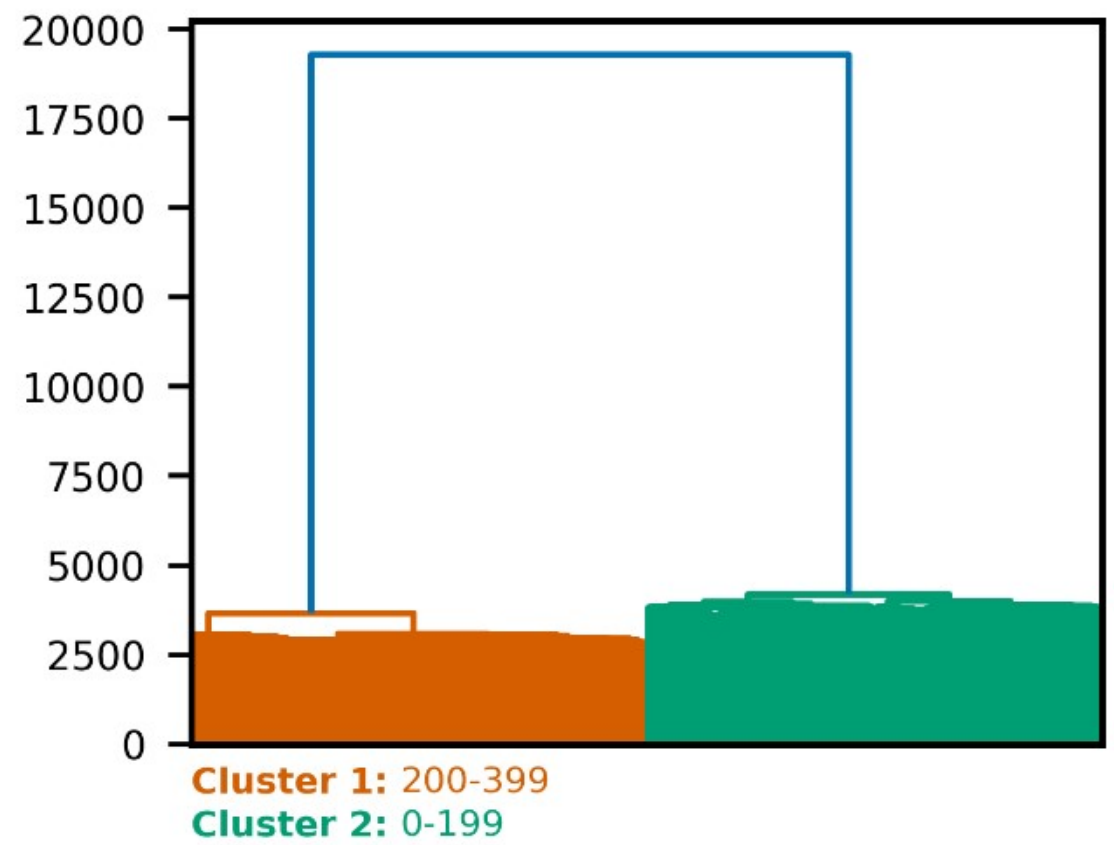

Intra; No Sampling

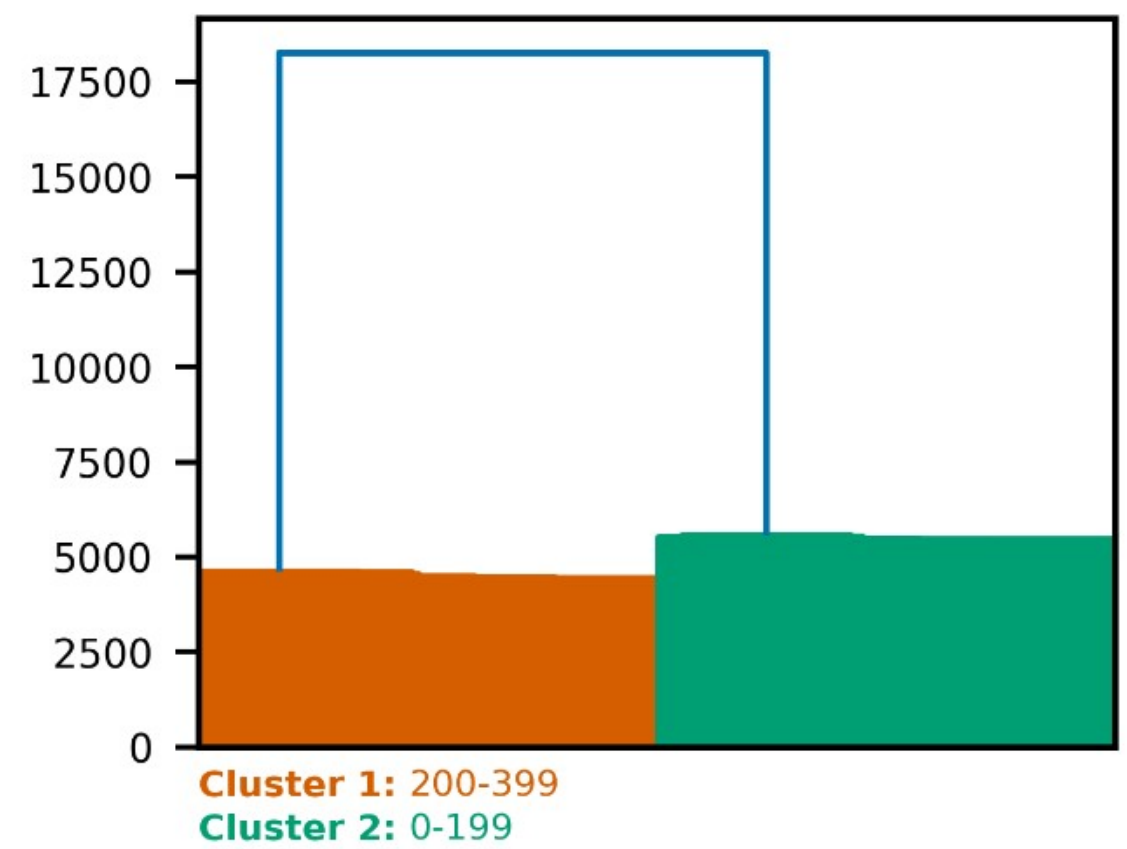

Intra; 50% Diversity Sampling

Figure S12. Adenylate kinase pathway dendrograms for SHINE intra (with and without sampling).

Table S1. Population distribution analysis for the HIF-2α system, comparing the population percentages of the two dominant clusters obtained using the SHINE intra scheme (with and without 50% sampling) and the three proposed PRISM variants at different cluster counts (k).

Method	Sampling	k	Cluster 1 Population (%)	Cluster 2 Population (%)
**SHINE intra**	None	-	52.0	48.0
	Quota	-	52.6	47.4
	Diversity	-	51.7	48.3
**PRISM Option 1**	None	6	51.1	48.9
		10	52.7	47.3
		20	53.4	46.6
		50	53.4	46.6
		80	53.4	46.6
**PRISM Option 2**	None	6	53.4	46.6
		10	53.4	46.6
		20	53.4	46.6
		50	53.4	46.6
		80	53.4	46.6
**PRISM Option 3** $k_{\text{final}} = k$	None	6	53.8	46.2
		10	53.4	46.6
		20	53.4	46.6
		50	53.4	46.6
		80	53.4	46.6
**PRISM Option 3** $k_{\text{final}} = 2k$	None	6	53.4	46.6
		10	53.5	46.5
		20	53.4	46.6
		50	53.4	46.6
		80	53.4	46.6

Table S2. Comparison of clustering times (in seconds) across benchmark processes using the SHINE intra scheme (with and without 50% sampling) and the three proposed PRISM variants at different cluster counts (k). Runtime scaling benchmarks were performed on standard CPU hardware, and wall-clock times reflect end-to-end clustering runtimes.

Method	Sampling	k	ALA	AdK	HIF-2α
**SHINE intra**	None	-	0.170	10.228	407.385
	Quota	-	0.172	6.245	256.250
	Diversity	-	4.862	24.678	3994.310
**PRISM Option 1**	None	6	0.466	6.464	130.244
		10	0.160	6.987	132.350
		20	0.155	9.394	137.403
		50	0.169	14.789	143.507
		80	0.184	17.599	147.360
**PRISM Option 2**	None	6	0.207	7.736	139.126
		10	0.229	8.258	141.912
		20	0.248	11.285	154.182
		50	0.234	39.028	214.980
		80	0.232	84.886	288.977
**PRISM Option 3** $k_{\text{final}} = k$	None	6	0.217	7.029	136.678
		10	0.227	7.130	135.875
		20	0.227	7.774	137.203
		50	0.250	12.004	149.234
		80	0.258	14.600	153.326
**PRISM Option 3** $k_{\text{final}} = 2k$	None	6	0.216	7.433	135.153
		10	0.231	7.732	141.489
		20	0.240	8.773	141.890
		50	0.274	12.906	148.802
		80	0.278	15.254	155.526
